# Duration Is Not a Reliable Indicator for Anticipating Event Boundaries

**DOI:** 10.1007/s42113-025-00243-x

**Published:** 2025-04-08

**Authors:** Viviana Sastre Gomez, Rebecca Defina, Paul Garrett, Jeffrey M. Zacks, Simon Dennis

**Affiliations:** 1 Melbourne School of Psychological Sciences, 700 Swanston Street, Carlton, VIC 3053 Australia; 2 Department of Psychological and Brain Sciences, One Brookings Drive, St. Louis, MO 63105 USA

**Keywords:** Event duration, Smartphone data, Sampling methods, Cognition

## Abstract

Research in event cognition has shown that how people segment events plays an important role in how they are perceived and remembered. One potential source of information for anticipating upcoming event boundaries is the typical duration of the event. The small amount of prior work that exists suggests that the durations of daily events have normal distributions and that people have accurate prior beliefs about these durations (Griffiths & Tenenbaum, 2006). This research study aims to further explore the temporal distribution of self-reported events from daily life. Forty-eight participants provided information regarding the duration of events from their daily life for 14 days. Event durations for various activity types were characterised using duration modelling with truncated normal, exponential, and gamma models. Predictions based on mean and median values, including ± 5% and ± 10% error margins, were obtained, and the cumulative hazard functions were derived. Exponential and gamma distributions fit most event categories better than the truncated normal distribution. Mean-based predictions of boundaries based on duration showed only 4–5% accuracy, indicating limited usefulness. This aligns with the cumulative hazard results, where most events followed the exponential distribution, suggesting no optimal time for predicting the end of the event. This suggests that when daily events are studied in an ecological context, most of them have little sign of a typical duration. Consequently, it is unlikely that duration estimation plays a large role in the anticipation of event boundaries.

**Supplementary Information:**

The online version contains supplementary material available at 10.1007/s42113-025-00243-x.

## Introduction

Even though our lives are filled with constant, continuous activity, we tend to describe activity in terms of discrete event units. The segmentation of ongoing activity into meaningful units is a normal concomitant of perception, comprehension, and memory (Zacks et al., 2007). These event units are not given by the physical structure of activity alone; rather, event segmentation is an active cognitive process that determines how individuals segment their stream of experience. In the words of Schwartz (2008, p. 54), “Events are not simply out there and ready-made, waiting to be seen, recognised, or described; they are what we make of them.”

In recent decades, research on event boundaries has largely focused on segmentation, shedding light on the mechanisms underlying boundary delineation and the dynamic updating of ongoing events (e.g. Doherty & Smeaton, 2008; Franklin et al., 2020; Kumar et al., 2023; Reynolds et al., 2007; Zacks et al., 2007; Zwaan et al., 1995). Within this literature, some studies have found that people can anticipate upcoming event boundaries. For instance, Zacks and colleagues (Zacks et al., 2011) found that when participants were asked to predict what would happen 5 s after a pause in a video, not only were they less accurate just before an event boundary, but they were also less confident, suggesting that they may be aware the event would change soon. Anticipation of event boundaries is also seen in studies utilising the dwell time paradigm (e.g. Hard et al., 2006; Hard et al., 2011; 2019; Kosie & Baldwin, 2019). In these studies, participants watch events unfold through self-paced progression through a series of still images. The time they dwell on each image before clicking through to the next increases leading up to a boundary, peaks at the boundary, and then decreases again. Baldwin and Kosie (2021) use this quadratic dwell time pattern to claim that people must be anticipating event boundaries and that this anticipation influences sensitivity to reactive cues for boundary detection.

There are at least four ways by which individuals could anticipate an upcoming event boundary:

1. It is possible that the nature of the perceptual stimulus itself changes in the lead up to an event boundary; for instance, the rate of feature change may increase. Indeed, the rate of pixel change shows the same quadratic pattern around event boundaries as dwell time. However, dwell time tends to start increasing well before pixel change (Hard et al., 2011), suggesting there may be more motivating boundary anticipation than the physical nature of the event.

A defining characteristic of the Feature Change Model is its reliance on perceptual stimuli—such as visual, auditory, or spatial cues—to signal the end of an event. Changes in these perceptual features establish where boundaries occur, indicating that this mechanism operates primarily through bottom-up processes and does not depend on prior knowledge of the event. The model is based on the simple expectation that perceptual features will remain consistent until a boundary occurs.

2. People could be anticipating event boundaries based on knowledge of the goal and event structure (Baldwin et al., 2001; Bower, 1982; Saylor et al., 2007; Zacks, 2004; Zacks & Tversky, 2001).
3. Another possible way to anticipate an event boundary is entropy, or the uncertainty associated with predictions about what happens next. This mechanism, as proposed by Baldwin and Kosie (2021), suggests that people anticipate boundaries when uncertainty peaks. Recent work by Kumar et al. (2023) supports this view, showing that models incorporating uncertainty more closely match human segmentation patterns than those focusing solely on prediction accuracy. A key characteristic of the entropy mechanism is its reliance on pre-boundary information. Unlike models that depend on cues at or after the boundary, entropy exclusively uses information available beforehand, making it particularly suitable for both segmentation and anticipation tasks. Importantly, the uncertainty in the entropy model is informed by expectations generated based on knowledge of the event type, enabling the model to predict transitions effectively. By identifying moments of peak uncertainty in the flow of events, the entropy model effectively signals transitions, even in the absence of explicit perceptual changes. Notably, entropy has been a foundational element in computational models of event segmentation (e.g. Brand & Kettnaker, 2000; Chen et al., 2007).
4. Finally, another way people may anticipate event boundaries is the typical duration of events. For instance, when watching a movie at the cinema, people may anticipate that it is about to end based on their knowledge of typical movie durations thus anticipate when it is likely to end (c.f. time-based prospective memory, McDaniel and Einstein, 2007). This potential mechanism has received limited attention to date, but there is some evidence that duration information could be used as part of event boundary anticipation. Hanson and Hanson (1996) modelled observer’s segmentations using a recurrent neural network and found that the network’s learnt expectancies of event durations influenced its sensitivity to new perceptual information, much as Baldwin and Kosie (2021) suggest that uncertainty influences people’s sensitivity to change. It is this potential usefulness of duration information for event boundary anticipation that this paper aims to explore.

One reason duration has not been much investigated in studies of event individuation is that almost all event segmentation research has been carried out on a limited time-scale of seconds to minutes. This restriction reflects the methodological constraints of lab-based experimental research, as well as theoretical proposals that there is a cognitive mechanism that is specialised for the segmentation of events on time-scales from seconds to tens of minutes (Zacks et al., 2007). However, events can span broad temporal scales, from seconds (e.g. pouring a cup of tea) to hours (e.g. going for a hike). In this paper, we investigate the duration of conceptual event units outside of the laboratory, and thus potentially on longer time-scales within the course of a day.

People may anticipate event boundaries using two distinct timing strategies. One relies on external cues, such as clocks, to track elapsed time. For example, during a scheduled lecture, a student checks the clock and realises that the lecture should be ending soon. Since they already know the scheduled end time, checking the clock allows them to estimate how much time remains until it finishes.

In contrast, the other strategy depends on an internal sense of time (Allman et al., 2014; Buhusi et al., 2005), where prior experience with similar activities provides a reference for estimating when an event is likely to end. For instance, a person might anticipate the conclusion of a lecture not by checking the clock but by relying on their subjective perception of time progression combined with their knowledge of how long lectures typically last.

This ability to anticipate event boundaries based on internal timing is not limited to events with implicit time like lectures; it can also extend to activities without clear time reference. For example, when filling a bathtub, a person may develop an expectation of when it will be full based on past experiences rather than a precise time measurement. This study focuses primarily on duration-based segmentation rather than explicit clock-based timing, aligning more closely with how individuals anticipate event boundaries in the absence of external time cues.

The usefulness of duration information for event boundary anticipation crucially depends on the extent to which events have typical durations and that people know them. In certain events, time itself becomes a crucial cue for anticipating event endings. This is clear in events which are explicitly time-based, such as holding a stretch for 2 min, but also less obviously in many other daily events. For example, when baking a cake, checking it every 5 min as it approaches the estimated total baking time demonstrates how time could be a critical determinant. In such instances, time acts as the primary indicator of the cake’s readiness, illustrating how sensitivity to temporal cues can aid in predicting the event’s end.

It is possible to consider two potential distinct models of the duration mechanism. The first model posits that an internal clock (Allman et al., 2014; Buhusi et al., 2005) establishes the onset of an event and then predicts its conclusion at a time based on the mean or median duration. The second model posits an accumulation process with a threshold. In this scenario, activation increases at a steady rate as it would in a sequential sampling process (Smith & Ratcliff, 2004). Anticipation of event termination increases as the accumulated activation approaches the threshold. The threshold could then be adjusted to reflect the typical duration of the event type.

In both these models, the accuracy with which people estimate typical durations is crucial for anticipating the end of events. In previous work, Griffiths and colleagues demonstrated that people are sensitive to the prior distributions of event durations. In Griffiths and Tenenbaum’s (2006) study, participants were asked to make judgments about numerical quantities of everyday phenomena, including the durations of two daily routines: baking times for cakes and watching a movie. Using a Bayesian model, they found that people’s beliefs regarding the durations of these events were Gaussian. In a second study, Lewandowsky et al. (2009) also asked participants to estimate the duration of baking a cake and watching a movie. They utilised a within-subject version of iterated learning. In addition, they analysed data at the individual level instead of the aggregate level. Their results were consistent with those of Griffiths and Tenenbaum (2006); the distributions of cake baking times and movie watching were normal.

According to the study results by Griffiths and colleagues (2006; 2009), models that depend on typical durations appear plausible, as the two events they investigated (baking a cake and watching a movie) displayed normal distributions; therefore, using the mean or median as an estimate of probable duration could be accurate much of the time.

**Fig. 1:**
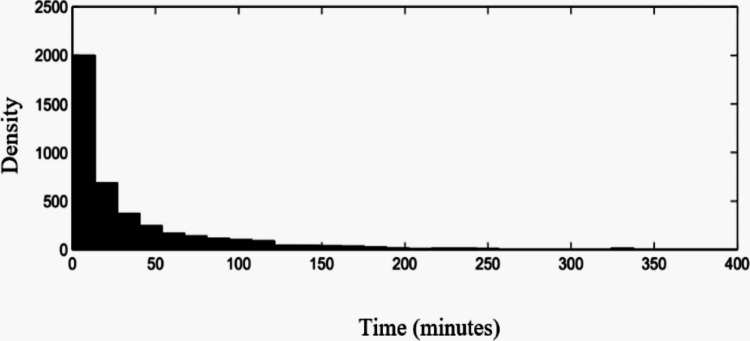
Daily activity distribution from Zhuang et al. (2012) data

**Fig. 2:**
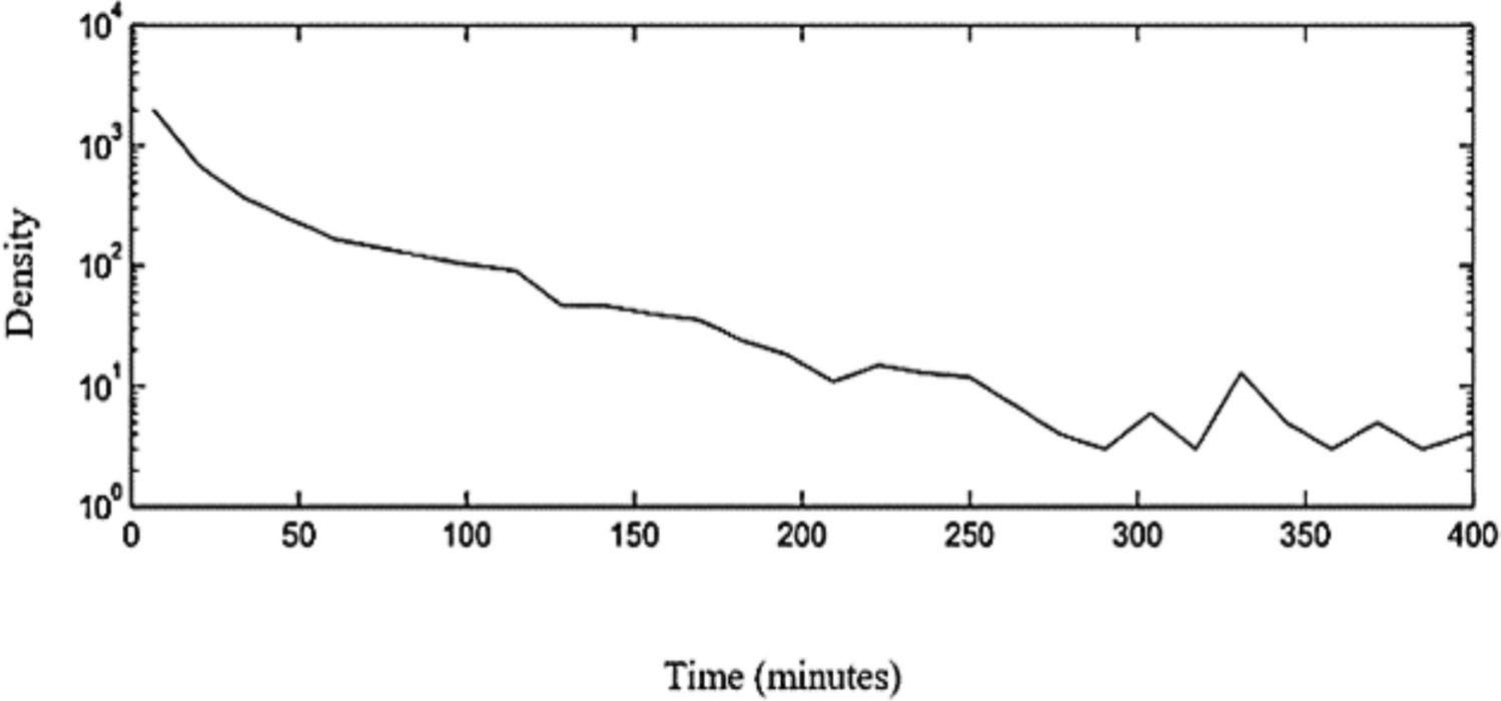
Event distribution histogram on a log scale from Zhuang et al. (2012) data

However, a study examining a broader range of daily life events found that event durations can have skewed distributions. Zhuang et al. (2012) utilised a lifelogging device and sensors to measure daily activities for not only seconds or minutes but also hours. They asked participants to wear Android smartphones to record GPS location, audio, and images as they went about their daily lives, for 4 weeks. They were also asked to divide their images into distinct events and to tag each episode with a set of labels each night. Figures 1 and 2 plot the event duration distribution using data from Zhuang et al. (2012) and illustrate that these event duration distributions are skewed rather than normal. Figure 2 illustrates a linear decline in event density as time progresses on a log linear plot, indicative of a pattern similar to exponential decay, where short-duration events occur frequently and longer events are increasingly rare. This pattern suggests that the majority of the probability mass is concentrated around shorter durations, and highlights a significant deviation from a normal distribution.

If event durations are highly skewed rather than normal then using the mean or median will be very inaccurate most of the time as there is very little probability mass around these values. In fact, the most probable value is very close to zero.

Furthermore, if the duration distributions are exponential then they have the memoryless property. That is, the probability that the end of the event will occur at any given point given that it has not already occurred is the same regardless of how much time has passed. Consequently, the amount of time that has passed could not be a reliable basis for anticipating the end of the event. In this case, the hazard function will be constant—or equivalently the cumulative hazard function will be a straight line (Klein & Moeschberger, 2003).

The observed differences in event duration distributions—normal in some cases and skewed in others—highlight that the assumption of normal duration distributions may not apply universally. Instead, these differences suggest the need to consider how methodological choices, such as event type granularity and reporting techniques, influence observed distributions. Griffiths and Tenenbaum (2006) and Lewandowsky et al. (2009) focused on specific event types (e.g. baking a cake or watching a movie), which are likely to have clearly defined duration expectations, and found normal distributions. In contrast, Zhuang et al. (2012) and Sreekumar et al. (2018) analysed a broader range of everyday activities without differentiating events by type, resulting in skewed distributions. This aggregation of event types may mask the distinct duration characteristics of individual categories. To address this gap, this paper aims to investigate the temporal duration of self-reported daily events using contemporary sampling techniques, distinguishing events by type to better understand how duration distributions vary across categories.

## Method

### Participants

Forty-eight participants over the age of 18 were recruited from two sources. The first source was a participant pool hosted at www.unforgettable.me. Unforgettable is a platform, similar to Mechanical Turk or Prolific Academic, for experience sampling that allows users to collect private data from their daily lives and make it available for researchers (Dennis et al., 2019). The second source consisted of various Facebook student groups, including International Students in Australia, International Students of Melbourne and Victoria, GRiPS (Graduate Researchers in Psychological Sciences), and International Students in Melbourne AU. Researchers posted weekly advertisements on these pages until the recruitment target was met, with each post containing study details and a contact email for research inquiries.

Participants were compensated between AUD $60.75 and AUD $97.50 based on the number of completed surveys. Five participants were excluded due to a lack of Wi-Fi; three withdrew, one was excluded because their phone was incompatible with the study’s requirements and two participants were excluded from the analysis due to their registered events consistently showing zero duration for over 90% of the observed period. The final sample comprised thirty-eight individuals between 21 and 64 years old (26 females and 12 males, mean age = 30.4). The participants in this study self-identified as members of diverse racial and ethnic groups: 14 respondents (37%) identified as Hispanic/Latino, 10 (26%) as White/Caucasian, 7 (18%) as Middle Eastern, 6 (16%) as Asian/Pacific Islander, and 1 (3%) as African. The bulk of participants (75%) were based in Australia, while the remaining participants were located in Canada (5%), Colombia (5%), Mexico (3%), Malaysia (3%), Singapore (3%), Sri Lanka (3%), and Vietnam (3%). All participants provided informed written consent. Information about the study and instructions were provided in English.

### Materials

**Fig. 3:**
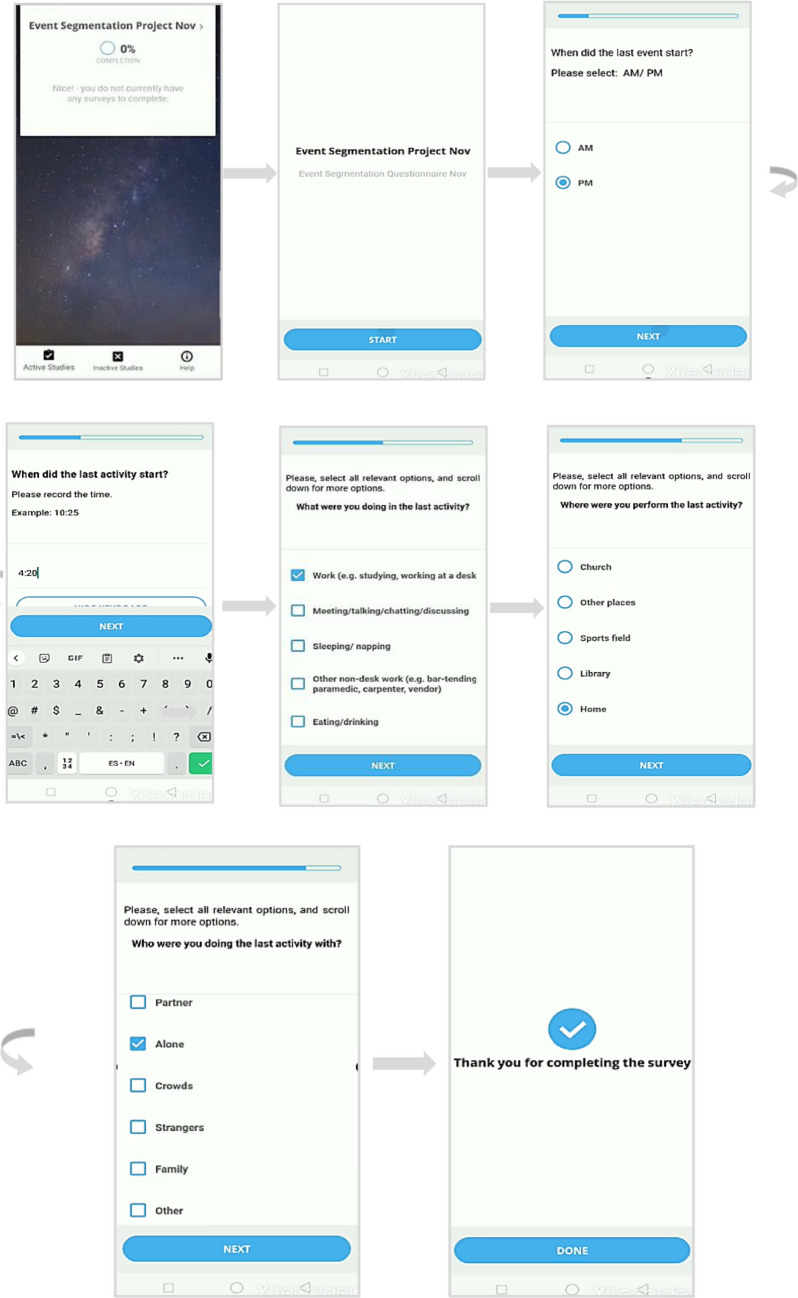
Event duration survey delivered through SEMA3

Micro-surveys were administered to participants as they went about their daily lives. Each survey asked four questions about the most recent event the participant had experienced: When did the event start? What sort of event was it? Where was the event located? and Who did you do the event with? Participants were instructed to complete the survey once they had completed their current task and to respond in relation to the most recent event (see Fig. 3, the survey conducted). Thus, the start time of the survey was taken to be the end time of the event, and the duration of the event was calculated as the difference between the survey time and the start time provided by the participant.

### Selecting Event Categories

Our questions and provided answer categories were based on preliminary research conducted by Hamm et al, 2012) wherein undergraduate participants were assigned to identify tags that effectively encapsulated the essence of episodes from their daily lives. For each event that they identified, they provided three tags. They were free to choose the tags as they wished, but were aware that these tags would be used for a later memory study in which they would be given two of the tags and be required to retrieve the remaining tag. Hamm et al (2012) found that tags related to activities, locations, and people were most helpful and clustered similar tags under categories within these domains.

In the study by Hamm et al. (2013), there were 15 categories under the activity domain, nine under the location domain, and six under the people domain. While piloting the current study, we noticed that participants were consistently using our free response “Other” options, particularly for locations and people. Consequently, we included additional categories to capture the most frequent of these tags. For instance, in the activity domain, six new tags were included: working/studying, other non-desk work, using social media, praying/meditating, sleeping/napping, and personal grooming/hygiene. Some categories from Hamm et al. (2013) were merged or redefined; for example, “giving a lecture,” “listening to a lecture,” and “using a computer” were consolidated under “working/studying,” and “walking” was included in the tag “exercising/playing sport/dancing/walking/running.” Additionally, we reviewed and removed rarely used or redundant categories, such as “tend to a baby” and “pick up/drop off.”

In the location domain, 11 more tags were added: work, library, park, gymnasium, garden, church, beach, farm, sports field, street, and transport. We also removed redundant categories such as “public places” and “outdoor” and consolidated specific tags; for example, “my office” and “other’s office” were merged under “Office,” and “classroom/meeting room” were consolidated under “school” tag. For the people domain, we included four additional tags: alone, classmates, pet(s), and partner.

This refinement process resulted in a final set of 15 categories in the activity domain, 17 in the location domain, and 10 in the people domain (see Table 1). This approach ensured that the categories better reflected the participants’ experiences without overcrowding the domains with infrequently used tags. Each question still included an “other” response option, which allowed participants to enter a free-text description. The order of options was randomised in each survey. Participants could select more than one option for the activity and people domains. However, they could only select one option for the location domain. For this study, we only considered and analysed the activity category.

**Table 1:** Event segmentation survey categories

Categories	Tags
Activity type (15)	- Watching movies/TV/listening to a concert/other performance - Exercising/playing sport/dancing/walking/running - Reading/writing - Eating/drinking - Working/studying - Other non-desk work (e.g. bar-tending, paramedic, carpenter, vendor) - Meeting/talking/chatting/discussing - Chores (cooking, cleaning, laundry) - Transiting (drive/fly/bus/taxi, other vehicles) - Shopping - Using social media - Praying/meditating - Sleeping/napping - Personal grooming/hygiene (e.g. brushing teeth, showering, doing hair) - Other activity
People (10)	Alone, family, friends, colleagues, classmates, pet(s), strangers, crowd, partner, other
Places (17)	Home, work, store, library, park, restaurant/café, office, gymnasium, garden, church, beach, school, farm, sports field, street, in transport (car/airplane/ship/truck and rail), other places

### Procedure

Participants were prompted to complete the survey using an ecological momentary assessment (EMA) application called SEMA3 (Koval et al., 2019). Seven survey notifications were semi-randomly distributed between 8:00 a.m. and 8:00 p.m. each day for 14 consecutive days. On average, survey notifications were separated from each other by 2 h. Following the notification, participants had around 80 min to complete each survey. Compliance was high: overall, participants responded to 91.45% of the surveys (*M* = 89.63 surveys, SD = 13.01).

### Data Analysis

We fit two different distributions to each activity type, as well as to the overall distribution. The first was a truncated normal distribution, which was included because some studies have shown that people believe that certain daily occurrences tend to have normal distributions (Griffiths & Tenenbaum, 2006). The distribution was truncated as the assumption is that any negative durations are mistakes, and they were eliminated from the data set, for model.

The second was an exponential distribution, which we chose to capture the skewed pattern found in the Zhuang et al. (2012) data. One conceptual peculiarity of choosing an exponential model is that it assumes that the most probable event duration is zero. Consequently, we incorporated a third model, the gamma model. This model can rise rapidly from zero but still has a generally skewed shape, addressing the distributional characteristics observed in our data.

To ensure comparability and minimise subjective bias, we employed weakly informative priors across all models and subdistributions. The truncated normal model (μ∼TNorm(400), σ∼TNorm(100)) was chosen to account for the observed peak around 400 min in the aggregate distribution while maintaining sufficient flexibility to allow for variation. The exponential model (λ∼Exp(1)) was used to represent right-skewed distributions without overly constraining the rate parameter. The gamma model (α∼Exp(2), β∼Gamma(1,0)) was selected for its ability to represent both skewed and peaked distributions. The consistent use of priors across subdistributions ensured a fair model comparison, reducing the need for case-specific justifications. These choices allowed the data to drive inference while maintaining realistic constraints and mitigating the risk of overfitting.

For each activity type, we fit three models as follows:

o Truncated Normal:
  $$\begin{array}{c}duration[i] \sim TNorm( \mu ,\sigma )\\ \mu \sim TNorm(400)\\ \sigma \sim TNorm(100)\end{array}$$
o Exponential:
  $$\begin{array}{c}duration[i] \sim Exp(\lambda )\\ \lambda \sim Exp(1)\end{array}$$
o Gamma:
  $$\begin{array}{c}duration[i] \sim Gamma(\alpha ,\beta )\\ \alpha \sim Exp(2)\\ \beta \sim Gamma(\mathrm{1,0})\end{array}$$

The estimation was conducted using the Nimble package in R, version 0.12.1 (NIMBLE Development Team, 2021). For each model, 10 chains of 101,000 MCMC samples were taken. The first 1000 samples of each chain were discarded. The widely applicable information criterion (WAIC) was used to select the best model (Watanabe, 2013). In addition, model convergence was evaluated visually using trace plots of the posterior chains (Depaoli and Schoot, 2017) and with R-hat (Moins, Arbel, Dutfoy, & Girard, 2022; Brooks & Gelman, 1998). All R-hat values should be close to 1, and values greater than 1.1 indicate that one or more chains have failed to converge for individual models. When a chain fails to converge, the draws returned by the sampler are not a sample from the posterior distribution and cannot be used for estimation (Brooks & Gelman, 1998).

To use a duration-based mechanism for anticipation, one must first determine the expected length of an event. We interpreted “expect” literally and assessed the effectiveness of central tendency measures in predicting event endings. While the mode is theoretically valid, it is uninformative in this context, as many observed distributions are exponential with modes of 0. As shown in Table 4, even when the distributions were best fit by a gamma distribution, the mode was 0 in all but one case. Consequently, we prioritised the mean and median, which provide more robust estimates for skewed distributions. Specifically, we evaluated the error percentage in predicting event durations within 5% and 10% margins of the mean and median across the entire dataset and for each activity type.

A second approach to analysing the potential to anticipate event boundaries based on duration involves using the cumulative hazard function, derived non-parametrically, for all data and each activity type. To compute the comparison of cumulative hazard functions for both the exponential and truncated normal distributions, it was necessary to estimate specific parameters. Initially, we fitted lines directly to the cumulative hazard function, which was appropriate when comparing only with the exponential distribution. However, incorporating the cumulative hazard function of the truncated normal distribution required knowledge of the mean and standard deviation of the underlying truncated normal distribution.

For this reason, we employed parameter estimates obtained through model fitting. Since this approach was necessary for the truncated normal distribution, we applied the same method to the exponential distribution. This methodology enabled a comparison of whether the cumulative hazard function for each event type aligned more closely with the exponential or the truncated normal distribution, ensuring a rigorous evaluation of distributional fit while introducing an inherent dependency on parametric assumptions.

## Results

We initially collected 3371 events. However, 153 events were excluded due to missing data (*n* = 58), negative durations (*n* = 13), zero durations (*n* = 76), and durations over 700 min (*n* = 6). These exclusions account for 2.8% of the total data. As a result, the final dataset comprised 3218 events.

In terms of the activity domain, 3958 activities were collected. We decided to exclude activities with few data points to ensure statistical reliability. Specifically, we excluded “Praying/meditating” (*n* = 33) due to its limited sample size. Additionally, we removed the “Other activities” tag (*n* = 207) because it encompassed a variety of events, and “Sleeping/napping” (*n* = 230) on the basis that participants were unable to anticipate event boundaries during sleep. As a result, the final dataset comprised 3488 activities, which is higher than the total number of events because participants were allowed to select more than one activity for the same event (see Table 2 for the breakdown of activity types).

**Table 2:** Breakdown of daily activities

Activities	*n*
Watching movies/TV/listening to a concert	330
Using social media	294
Eating/drinking	583
Working/studying	768
Meeting/talking/chatting	466
Chores	225
Personal grooming/hygiene	151
Shopping	96
Exercising/playing sport	135
Reading/writing	113
Transiting	240
Other non-desk work	87

Table 3 shows the WAICs for the truncated normal model and exponential model for each activity. Bolded results indicate the best fitting model. When the differences between models were less than five, we have bolded both results as the data does not clearly distinguish them. Following Gelman et al. (2014), Burnham and Anderson (2004), and Vehtari et al. (2017), WAIC differences below five are not considered meaningful because they may arise from small fluctuations in the data rather than true model differences.

**Table 3:** WAIC score for truncated normal, exponential, and gamma distribution models

	Truncated normal model	Exponential model	Gamma model
All data	36,216	35,016	**34,992**
Watching movies/TV/listening to a concert	3613	**3476**	**3475**
Using social media	3203	**3088**	**3091**
Eating/drinking	6076	**5965**	**5967**
Working/studying	8599	**8473**	**8468**
Meeting/talking/chatting	5102	**4947**	**4948**
Chores	2320	**2241**	**2244**
Personal grooming/hygiene	1579	**1557**	**1561**
Shopping	1060	**1033**	**1035**
Exercising/playing sport	1455	**1438**	**1442**
Reading/writing	**1199**	1218	1217
Transiting	**2448**	2454	2455
Other non-desk work	1028	**1015**	**1018**

*Note:* Numbers in **bold** indicate the better-fitting model (lower WAIC). Differences of less than five were not interpreted as meaningful

**Fig. 4:**
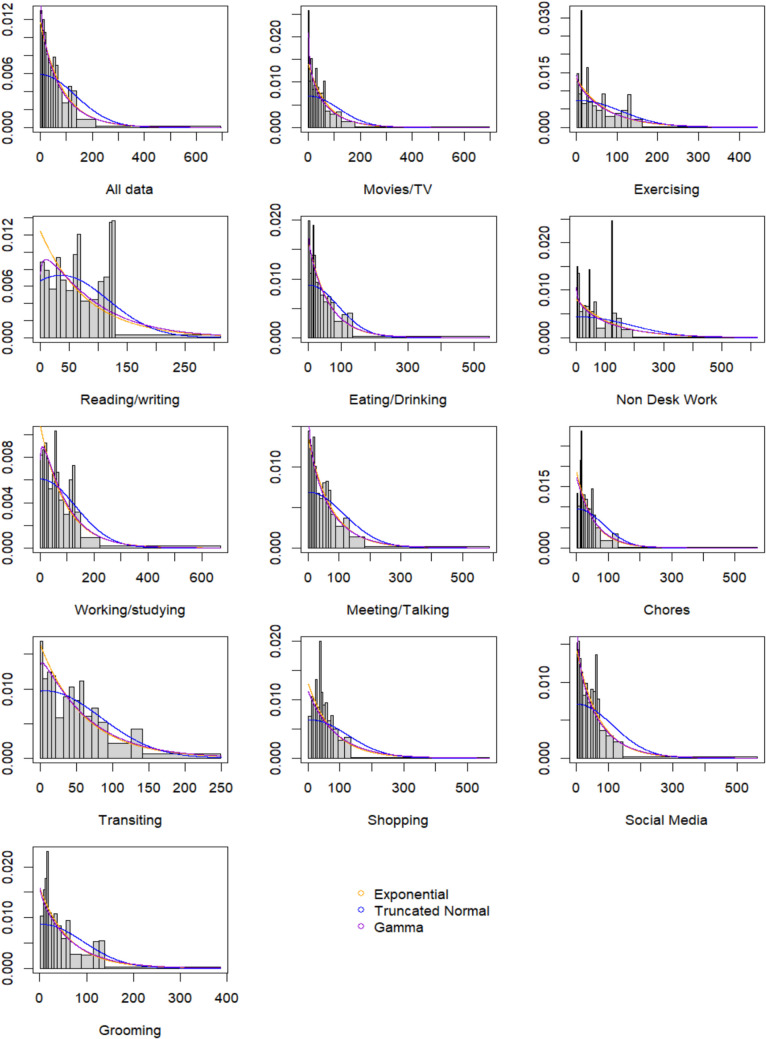
Vincentized histogram of the durations of events measured in minutes

Table 3 reveals that the exponential model was preferred not only for the aggregated data but also for most of the individual activity types, suggesting that these event types are skewed (see Fig. 4 for the fits and vincentized histograms for each activity type). The parameters of the truncated-normal and exponential models, along with their 95% confidence intervals, appear in Appendix A.

**Fig. 5:**
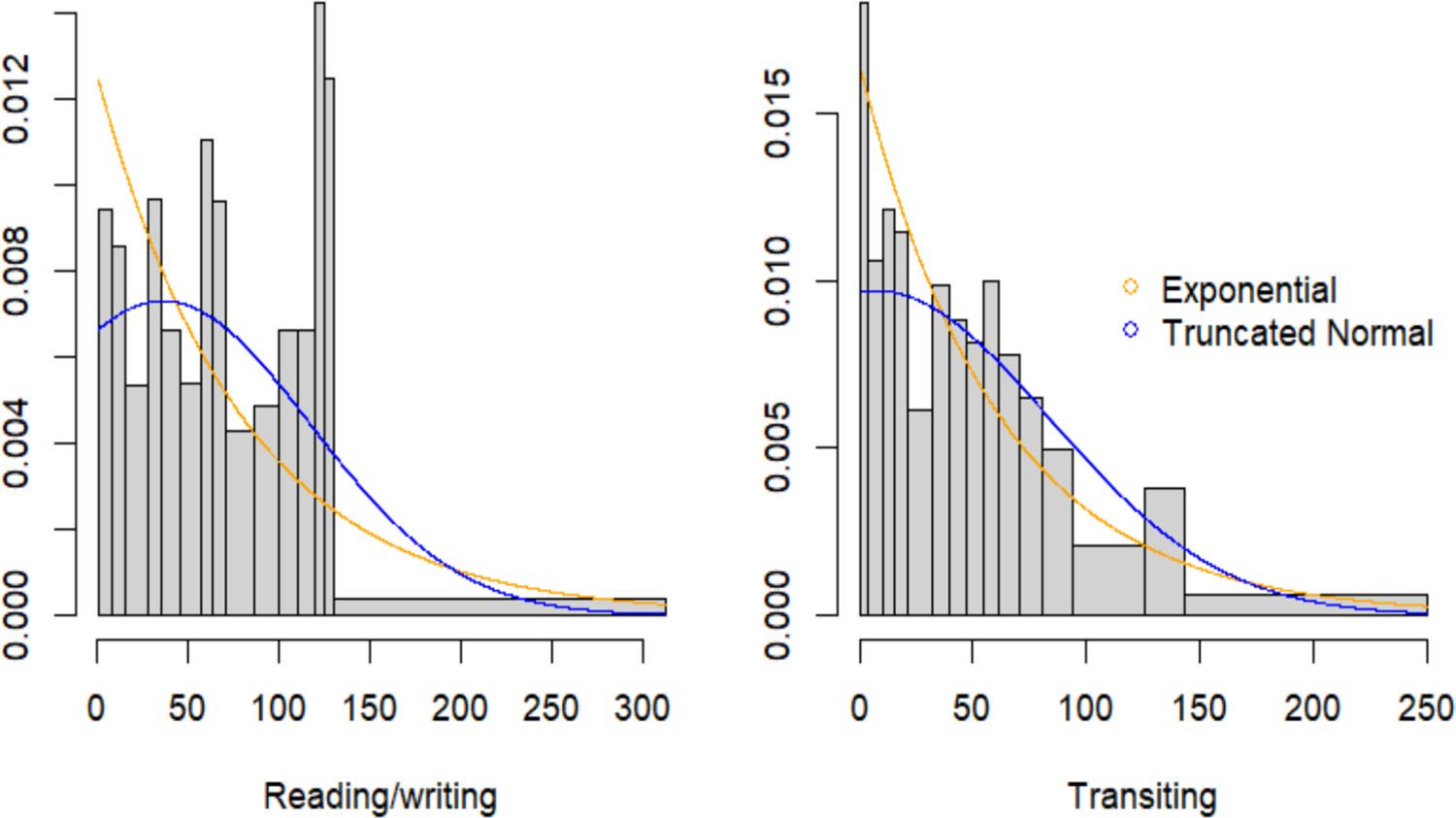
Vincentized histogram of the durations of events measured in minutes by exponential and truncated normal distribution

Figure 5 shows the histogram of duration values for the two events that fit the truncated normal better than the exponential: reading/writing and transiting. The orange line represents the fit of the exponential model using the posterior mean of lambda (λ), while the blue line represents the fit of the truncated normal model using the posterior means of mu (μ) and sigma (σ). Although the truncated normal distribution provided a better fit in both cases, it was not a good fit in either.

One might be concerned that the exponential distribution is a poor choice for comparison given it assigns high probability to zero length events. Consequently, we also tested the gamma distribution, focusing only on those activities for which the exponential was chosen in the previous exercise. The WAICs for the exponential and gamma models for each activity are presented in Table 3.

Table 3 shows that the gamma model was preferred over the exponential model in one case only for the aggregated data. Additionally, 10 out of 12 event type categories did not exhibit a clearly dominant pattern for either model, making it difficult to distinguish between the exponential and gamma distributions.

While the gamma model can capture early peaks, it is essential to note that when the alpha parameter is less than one, the mode of gamma distribution is zero. Our analyses revealed that the α parameter was less than one in all but one case. However, even when the modal duration was greater than zero (working/studying), it was still quite brief (see Table 4). These values represent the modes for those activity types where the gamma model was the most likely fit.

**Table 4:** Mode of gamma models

	Gamma model mode (minutes)
All data	0
Watching movies/TV/listening to a concert	0
Using social media	0
Eating/drinking	0
Working/studying	8.67
Meeting/talking/chatting	0
Chores	0
Personal grooming/hygiene	0
Shopping	0
Exercising/playing sport	0
Other non-desk work	0

**Fig. 6:**
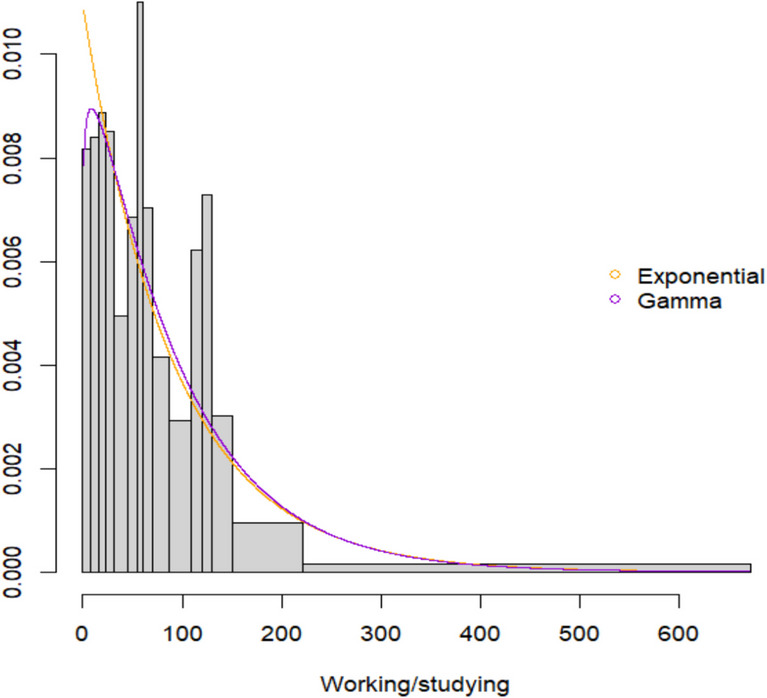
Vincentized histogram of the durations of events measured in minutes by exponential and gamma distribution

An example of the fit of a gamma model can be seen for the working/studying, in Fig. 6; the orange line represents the fit of the exponential model with the mean lambda (λ), and the magenta line represents the fit of gamma model with the mean of alpha ($\alpha$) and beta ($\beta$) (see all Vincentized histograms in Fig. 4).

To evaluate how effectively typical duration information could be used to predict event endings, we examined the accuracy of predictions based on mean and median event durations, an approach grounded in the internal clock mechanism (Allman et al., 2014; Buhusi et al., 2005). Table 5 presents data on the accuracy of predictions for the end of the event based on mean and median values, with margins of error set at 5% and 10% above and below these estimates. The results show that when predictions are based on the mean with a 5% margin of error, the probability of accurately predicting the event’s end does not exceed 10% for any event type or for the overall dataset. The results indicate that when predictions are based on the mean with a 5% margin of error, the probability of accurately predicting the event’s end does not exceed 10% for any event type or the overall dataset. The events with the highest accuracy using the mean were other non-desk work (8.0%), followed by chores (6.6%). The lowest accuracy was observed in reading/writing (1.7%), exercising/playing sports (2.2%), and meeting/talking/chatting (2.6%). For median-based predictions with a 5% margin of error, the events with the highest accuracy were transiting (5.8%) and using social media (5.7%), while the lowest were exercising/playing sports (0.7%) and eating/drinking (2.9%).

**Table 5:** Accuracy of event duration predictions using 5% and 10% above and below margins

	Mean		Median	
	5%	10%	5%	10%
All data	0.019	0.059	0.036	0.092
Watching movies/TV/listening to a concert	0.036	0.072	0.045	0.067
Using social media	0.042	0.084	0.057	0.078
Eating/drinking	0.053	0.077	0.029	0.060
Working/studying	0.026	0.069	0.032	0.106
Meeting/talking/chatting	0.045	0.081	0.047	0.087
Chores	0.066	0.115	0.049	0.071
Personal grooming/hygiene	0.026	0.066	0.046	0.052
Shopping	0.052	0.104	0.052	0.093
Exercising/playing sport	0.022	0.081	0.007	0.029
Reading/writing	0.017	0.061	0.035	0.115
Transiting	0.058	0.100	0.058	0.091
Other non-desk work	0.080	0.121	0.034	0.057

When the margin of error is increased to 10% for mean-based anticipation, only three out of twelve event types exceed the 10% accuracy threshold: other non-desk work (12.1%), chores (11.5%), and shopping (10.4%). For median values with a 10% margin of error, the events achieving over 10% accuracy in anticipating the event’s end are reading/writing (11.5%) and working/studying (10.6%).

Additionally, to analyse the accumulation model—which suggests that activation builds up gradually until it reaches a threshold based on the typical duration—we calculated the cumulative hazard function. A constant cumulative hazard function, indicated by a straight line, would suggest that the probability of the event ending at any given moment remains constant, regardless of how much time has passed—implying that there would be no basis for setting the threshold in the model.

**Fig. 7:**
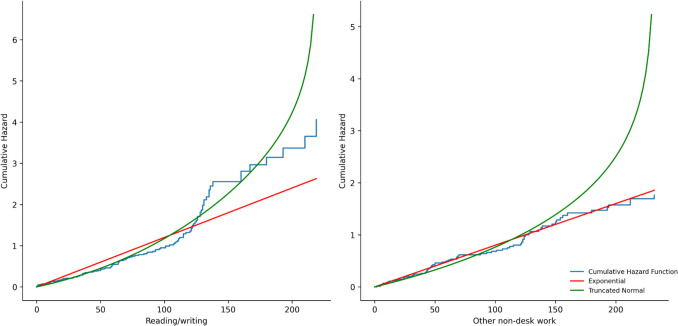
Cumulative hazard analysis: event termination patterns over minutes

The cumulative hazard analysis results indicate that most event types demonstrate consistent patterns in their hazard functions, illustrated graphically by a straight line with a uniform slope, closely approximating the cumulative hazards characteristic of an exponential distribution, except for three event types—transiting, reading/writing, and working/studying—that do not conform to this pattern and instead show that deviate more from the exponential cumulative hazard model (see Appendix B—Supplementary Materials). The alignment between the cumulative hazard analysis and the expected exponential model fitting indicates that, for most event types, the observed hazard functions correspond well with the assumptions of the exponential model. Such alignment strengthens the validity of the model’s predictions regarding event duration and threshold behaviour. Figure 7 provides an example of these cumulative hazard functions: the blue lines represent “reading/writing” and “other non-desk work” event types, the red line illustrates the exponential model, and the green line represents the truncated normal model. The “other non-desk work” event type aligns closely with the red line, suggesting it follows an exponential pattern without reaching a critical threshold that would indicate the event’s end. Conversely, the “reading/writing” activity type shows a wobbly pattern, diverging from the straight line of the exponential model.

## Discussion and Conclusion

People are capable of identifying that events are about to finish (Zacks et al., 2011; Hard et al., 2006; Hard et al., 2011; 2019; Kosie & Baldwin, 2019). Four theoretical mechanisms could explain how people anticipate these boundaries: (1) the anticipation of perceptual changes in stimuli (Hard et al., 2011), (2) knowledge of event goals and structures (Baldwin et al., 2001; Bower, 1982; Saylor et al., 2007; Zacks, 2004; Zacks & Tversky, 2001), (3) entropy or uncertainty associated with predictions about what will happen next (Brand & Kettnaker, 2000; Chen et al., 2007; Kumar et al., 2023), and (4) the typical duration of events, which aligned with the context of time-based prospective memory (McDaniel & Einstein, 2007). In this study, our primary aim was to explore the duration of daily events reported by people and to understand the underlying distribution of these durations in order to evaluate the possibility that people might use information about event durations to anticipate the end of the event. This possibility crucially depends on the extent to which events have typical durations and whether people are aware of these durations (see Griffiths and Tenenbaum., 2006; Lewandowsky et al., 2009).

Two distinct models could explain this potential duration mechanism for event boundary anticipation. The first model proposes that an internal clock (Allman et al., 2014; Buhusi et al., 2005) predicts event endings based on the mean or median duration, such as estimating when a cake will be ready by referencing typical baking times. The second model is an accumulation model with activation reaching a predefined threshold, which is adjusted to the typical duration of the event type (Smith & Ratcliff, 2004).

Our findings suggest that duration-based anticipation of event boundaries may not be a broadly applicable mechanism, particularly in broader cases where event types are not as narrowly defined as in studies like Griffiths and Tenenbaum (2006). Exponential and gamma distributions fitted most event types better than the truncated normal, indicating a lack of clear, typical durations. This is consistent with patterns observed in studies by Zhuang et al. (2012) and Sreekumar et al. (2018), which also found that event durations tend to follow skewed exponential distributions. Nevertheless, some specific types of events with well-defined temporal structures—such as watching a film—may still allow for duration to serve as a reliable cue. Similarly, our results showed that anticipating the end of events based on their mean or median was highly inaccurate, with mean-based predictions achieving only 4–5% accuracy, reinforcing the limited utility of duration information for most daily events. The cumulative hazard analysis also showed a uniform slope in the hazard function in the vast majority of cases (Klein & Moeschberger, 2003), indicating that events primarily followed an exponential pattern and did not present a distinct time point for anticipating their end.

However, not all event types conform neatly to an exponential pattern. Three event types—reading/writing, transiting, and working/studying—deviated from this trend. For reading/writing and transiting, the WAIC scores indicated that the exponential model did not fit well (see Table 3). Additionally, the cumulative hazard analysis for reading/writing showed an irregular pattern, with a sharp increase in the probability of the event ending as accumulated activation approached a critical level. This suggests that the termination threshold could be adjusted to reflect the typical duration of this event type, as seen in the change in the cumulative hazard line around the two-hour mark (see Fig. 7). However, when using the mean or median to predict the end of these events, the estimates were not reliable; predicting the end of reading/writing within 10% of the median duration resulted in an 88.5% probability of error, and for transiting, the error probability was 90.9% (see Table 5). This highlights the low accuracy of using central tendency measures to anticipate when an event will end, even in cases like reading/writing and transiting, which deviate from the exponential distribution.

Regarding the third event type, working/studying, the WAIC scores showed no clearly dominant pattern for either the exponential or gamma models, reflecting ambiguity between the two. Although the gamma model indicated a non-zero mode for working/studying (8.67 min, see Table 5), this duration remains brief and does not establish a robust pattern. In light of the above, although the exponential model was not favored for these three event types (reading/writing, transiting, and working/studying), it is not conclusive that events deviating from the exponential distribution and displaying more unstable cumulative hazard rates can use duration as a cue to anticipate their end, even in cases where the distributions do not fit well with an exponential model.

Although studies like those by Griffiths and Tenenbaum (2006) and Lewandowsky et al. (2009) suggest that time may be a potential indicator for predicting when an event might end, our findings suggest that relying solely on temporal cues is practical for accurate anticipation. Specifically, using mean and median durations to predict upcoming events would prove inaccurate, as predictions based on these measures were incorrect over 90% of the time. This potential inefficiency is further supported by our analysis of the cumulative hazard function, which did not reveal a distinct peak that would indicate an optimal time for event termination across most event types. The consistency among the three analyses—cumulative hazard, mean/median duration accuracy, and WAIC outcomes—suggests that people could not use duration to anticipate event boundaries for the vast majority of daily events. This study aimed to explore the potential role of duration information in anticipating the end of daily events. However, our findings suggest that duration alone offers limited benefit for predicting event boundaries across many typical event types. Nevertheless, it is important to acknowledge that there are specific scenarios where duration may still serve as a useful cue, such as baking a cake or other activities with consistent temporal structures. While event duration may not be a broadly applicable cue, its role in such specific contexts warrants further exploration.

Future research should also consider how the level of analysis influences duration patterns. Aggregated datasets, like those in Zhuang et al. (2012), and granular approaches, like Griffiths and Tenenbaum (2006), offer complementary insights into event cognition. Bridging these perspectives by systematically varying granularity could illuminate whether skewness in duration distributions is a product of aggregation or an inherent characteristic of daily activities.

Beyond statistical patterns, another factor that may contribute to variability in duration estimations is memory for event start times. An additional factor worth considering is the potential influence of participants’ memory for event start times on the observed distributions of durations. Unlike prior experiments by Griffiths and Tenenbaum (2006) and Lewandowsky et al. (2009), where participants were prompted for duration information, our study required participants to rely on memory for the start times of events. This reliance could mean that our participants’ estimates were a convolution of the actual event duration and memory error. Memory for start times might introduce substantial variability in participants’ estimates of event duration, particularly for longer or less salient events.

This limitation highlights an important avenue for future research. Specifically, understanding whether participants’ ability to extract a “typical duration” for an event type is more influenced by memory for the event duration (as encoded during or after the event) than by the actual duration is a critical question. This raises an important question about how participants’ memory of event durations might influence their ability to segment events into categories with typical durations, rather than relying solely on actual durations. Future studies could address this by comparing performance in tasks where participants have access to ongoing duration information versus tasks that require retrospective estimation of duration.

However, it is also important to consider that duration may not act in isolation when people anticipate event boundaries. Other mechanisms—such as entropy, knowledge of goals, and perceptual changes—could interact with duration-related cues to provide a more reliable foundation for event boundary anticipation in daily life. Investigating these alternative mechanisms alongside variations in granularity could yield a more comprehensive understanding of how humans anticipate and segment events.

## Supplementary Information

Below is the link to the electronic supplementary material.

Supplementary file1 (PDF 74 KB)

Supplementary file2 (PDF 309 KB)

## Data Availability

Data is provided within the supplementary information files, and all data, materials, code, and appendix are also available online in https://osf.io/ne329/?view_only = fdaa0a5133894b128a5aade22 df29c51.

## Declarations

### Open Access

All data, materials, code, and appendix are available online. https://osf.io/ne329/?view_only=fdaa0a5133894b128a5aade22df29c51.
