# Supplementary material for: Duration Is Not a Reliable Indicator for Anticipating Event Boundaries
Source: Comput Brain Behav. 2025 Apr 8;8(4):553–67. doi: 10.1007/s42113-025-00243-x (PMC13298632; doi:10.1007/s42113-025-00243-x)
Supplement: Supplementary file 1 — Supplementary file1 (PDF 74 KB) [file 42113_2025_243_MOESM1_ESM.pdf]

Appendix A

Parameters and 95% Confidence Intervals for Truncated Normal, Exponential, and Gamma Models

	Truncated Normal Model				Exponential Model		Gamma Model			
	μ		σ		λ		α		β	
All Data	0.246	(0.006, 0.904)	134.95	(131.70, 138.29)	0.012	(0.011, 0.012)	0.892	(0.854, 0.930)	0.010	(0.010, 0.011)
Watching movies/TV/listening to a concert	1.878	(0.047, 6.808)	113.94	(105.54, 123.18)	0.014	(0.013, 0.016)	0.834	(0.728, 0.948)	0.011	(0.009, 0.013)
Using social media	2.123	(0.055, 7.644)	110.74	(102.10, 120.27)	0.014	(0.013, 0.016)	0.891	(0.772, 1.020)	0.012	(0.010, 0.015)
Eating/drinking	1.362	(0.036, 4.884)	87.91	(82.96, 93.20)	0.016	(0.015, 0.018)	0.971	(0.876, 1.071)	0.015	(0.014, 0.018)
Working/studying	1.684	(0.044, 6.089)	129.77	(123.39, 136.57)	0.011	(0.010, 0.012)	1.103	(1.008, 1.203)	0.012	(0.011, 0.013)
Meeting/talking/chatting	1.559	(0.041, 5.665)	114.38	(107.24, 122.09)	0.014	(0.012, 0.015)	0.894	(0.797, 0.997)	0.012	(0.010, 0.014)
Chores	2.269	(0.059, 8.055)	81.55	(74.27, 89.70)	0.019	(0.016, 0.021)	1.000	(0.845, 1.169)	0.017	(0.014, 0.021)
Personal grooming/hygiene	5.434	(0.149, 18.343)	87.76	(77.78, 98.92)	0.016	(0.013, 0.019)	0.957	(0.779, 1.154)	0.014	(0.010, 0.017)
Shopping	7.758	(0.218, 26.286)	115.71	(99.82, 134.35)	0.012	(0.010, 0.016)	0.993	(0.766, 1.254)	0.011	(0.008, 0.015)
Exercising/playing sport	7.469	(0.212, 24.859)	102.41	(89.81, 116.46)	0.013	(0.011, 0.016)	0.943	(0.759, 1.150)	0.012	(0.009, 0.015)
Reading/writing	36.17	(3.186, 67.179)	81.29	(63.09, 101.78)	0.012	(0.010, 0.015)	1.138	(0.893, 1.416)	0.013	(0.009, 0.017)
Transiting	7.190	(0.223, 22.264)	76.41	(67.95, 84.75)	0.016	(0.014, 0.019)	1.044	(0.887, 1.216)	0.016	(0.013, 0.019)
Other non-desk work	16.01	(0.471, 52.001)	170.78	(145.05, 200.68)	0.008	(0.007, 0.010)	0.894	(0.680, 1.137)	0.007	(0.005, 0.009)

Note: Values are point estimates with 95% confidence intervals in parentheses, obtained via maximum likelihood estimation.
