# Supplementary figures and images for: Duration Is Not a Reliable Indicator for Anticipating Event Boundaries
Source: Comput Brain Behav. 2025 Apr 8;8(4):553–67. doi: 10.1007/s42113-025-00243-x (PMC13298632; doi:10.1007/s42113-025-00243-x)

## Appendix B

### Temporal Analysis of Cumulative Hazard Functions in Minutes

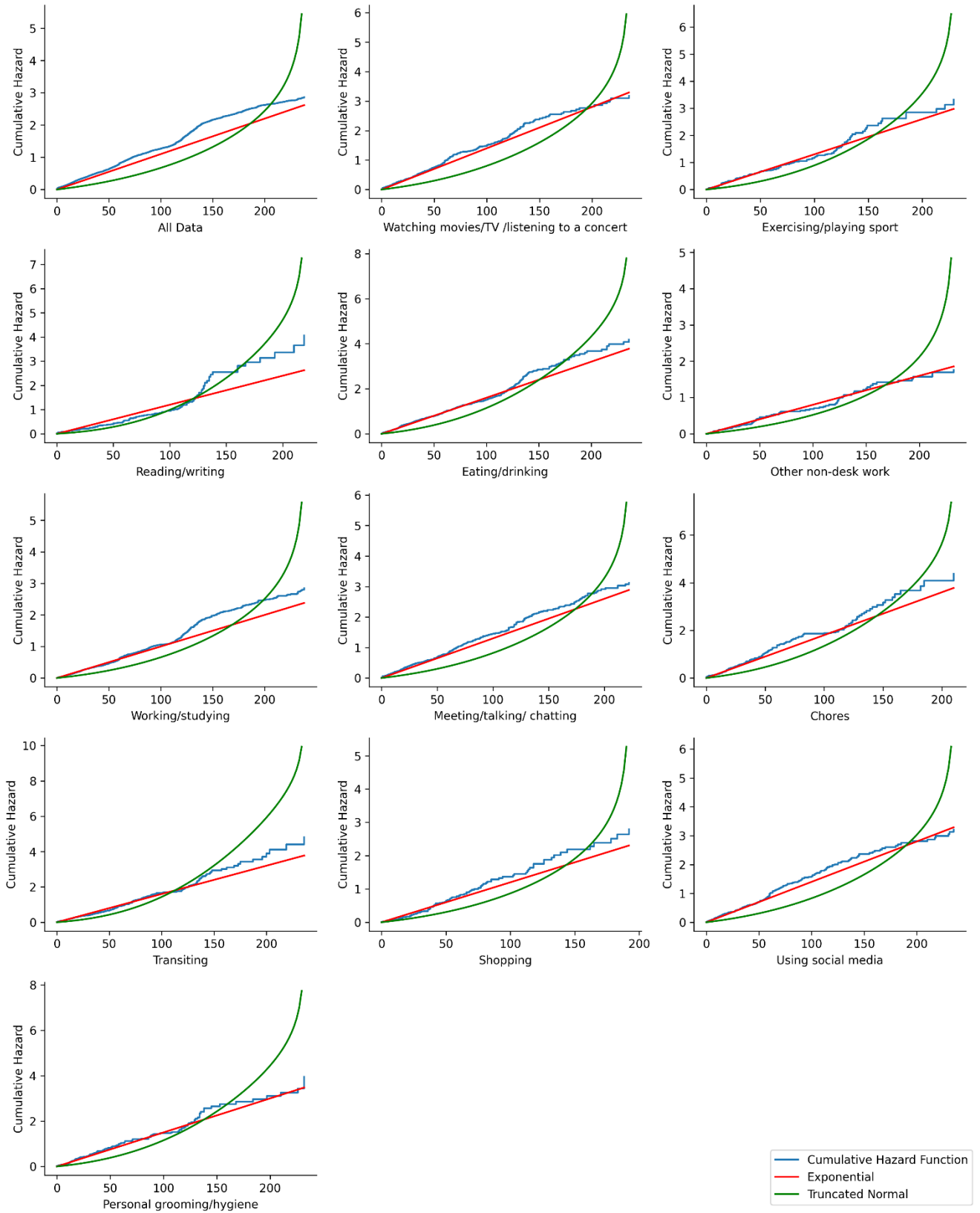

Supplement: Supplementary file 2 — Supplementary file2 (PDF 309 KB) [file 42113_2025_243_MOESM2_ESM.pdf]
